# 1754. Parainfluenza Virus Co-detection With Respiratory Syncytial Virus or Influenza is Associated with Higher Odds of Hospitalization in Children < 2 Years Old, New Vaccine Surveillance Network (NVSN), 2016–2020

**DOI:** 10.1093/ofid/ofad500.1585

**Published:** 2023-11-27

**Authors:** Yasmeen Z Qwaider, Annabelle De St. Maurice, Justin Z Amarin, Tess Stopczynski, Andrew J Spieker, Laura S Stewart, James Chappell, Eileen J Klein, Janet A Englund, John V Williams, Marian G Michaels, Elizabeth P Schlaudecker, Mary A Staat, Pedro A Piedra, Vasanthi Avadhanula, Jennifer E Schuster, Rangaraj Selvarangan, Heidi L Moline, Natasha B Halasa, Ariana Perez, Peter G Szilagyi, Geoffrey A Weinberg

**Affiliations:** Vanderbilt University Medical Center, Nashville, Tennessee; Los Angeles County Department of Health/UCLA, Los Angeles, California; Vanderbilt University Medical Center, Nashville, Tennessee; Vanderbilt University Medical Center, Nashville, Tennessee; Vanderbilt University Medical Center, Nashville, Tennessee; Vanderbilt University Medical Center, Nashville, Tennessee; Vanderbilt University Medical Center, Nashville, Tennessee; University of Washington School of Medicine, Seattle, Washington; Seattle Children’s Hospital, Seattle, Washington; University of Pittsburgh, Pittsburgh, Pennsylvania; UPMC Children's Hospital of Pittsburgh, Pittsburgh, Pennsylvania; Cincinnati Children's Hospital Medical Center, Cincinnati, Ohio; Cincinnati Children’s Hospital Medical Center, Cincinnati, Ohio; Baylor College of Medicine, Houston, Texas; Baylor College of Medicine, Houston, Texas; Children’s Mercy Kansas City, Kansas City, Missouri; Children’s Mercy Kansas City, Kansas City, Missouri; Centers for Disease Control and Prevention, Atlanta, Georgia; Vanderbilt University Medical Center, Nashville, Tennessee; CDC, Avondale Estates, Georgia; UCLA School of Medicine, Agoura Hills, California; University of Rochester School of Medicine & Dentistry, Rochester, NY

## Abstract

**Background:**

Parainfluenza virus (PIV) is an important cause of acute respiratory illness in children; however, scarce data have been reported on the clinical significance of PIV co-detection with other viruses.

**Methods:**

Between 12/01/2016 and 03/31/2020, we conducted active surveillance for children who presented to the emergency department or were hospitalized with fever or respiratory symptoms at seven U.S. medical centers within NVSN. Demographic and clinical data were collected through parent/guardian interviews and chart abstraction. Nasal and/or throat swabs were tested for PIV types 1–4; respiratory syncytial virus (RSV); rhinovirus or enterovirus (RV/EV); adenovirus (AdV); common cold coronaviruses (ccCoV) 229E, HKU1, NL63, and OC43; SARS-CoV-2; influenza (Flu) A, B, and C; and human metapneumovirus (hMPV). We used a generalized linear mixed-effects model with a logit link to compare the odds of hospitalization between children < 2 years old with PIV-only detection and those with PIV co-detection. Our analysis included age, underlying conditions, and preterm birth as fixed effects and study site as a random effect.

Table 1.

**Figure:**
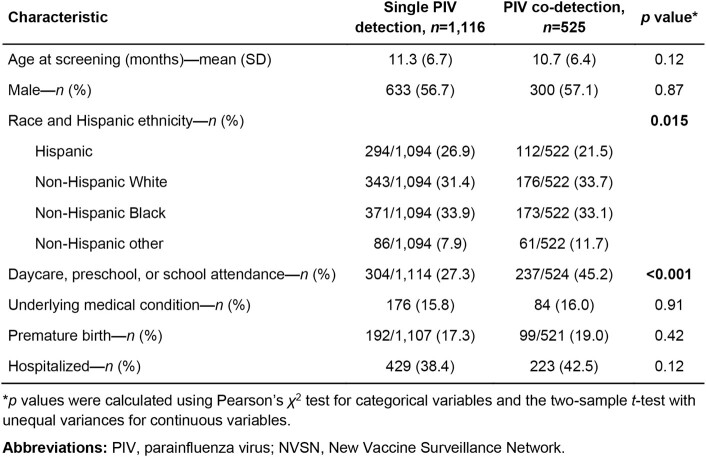

Comparison of the demographic and clinical characteristics of children with PIV-only detection and those with respiratory virus co-detection, NVSN, 2016–2020.

Figure 1.

**Figure:**
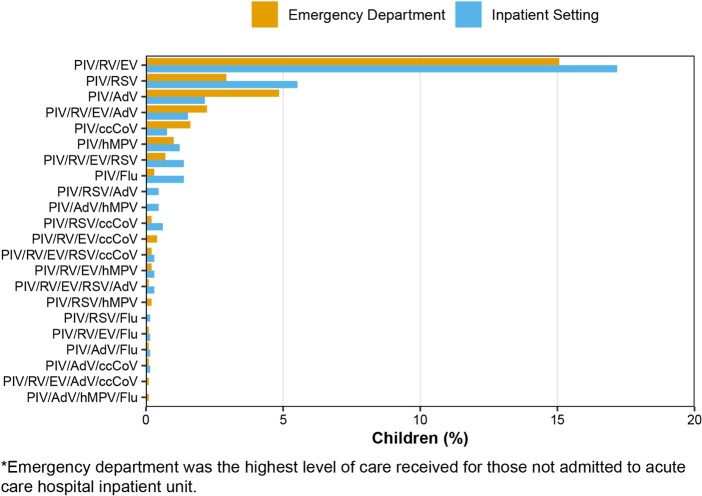

Relative frequencies of respiratory viruses co-detected with PIV in U.S. children <2 years old, stratified by highest* level of care received, NVSN, 2016–2020. Abbreviations: PIV, parainfluenza virus; RV/EV, rhinovirus or enterovirus; RSV, respiratory syncytial virus; AdV, adenovirus; ccCoV, common cold coronaviruses; hMPV, human metapneumovirus; Flu, influenza.

**Results:**

Of 17,850 children tested for PIV, 1,641 (9.2%) were positive: 1,116 (68.0%) with PIV-only detection and 525 (32.0%) with PIV co-detected with at least one other virus. The demographic and clinical characteristics of children with single PIV detection and those with co-detection are compared in **Table 1**. Notably, children with PIV co-detection were more likely to attend daycare, preschool, or school. Compared with PIV-only detection, the odds of hospitalization were higher for PIV/RSV (OR=2.18, 95% CI: 1.28–3.76, *p*=.004) and PIV/Flu (OR=5.61, 95% CI: 1.55–26.60, *p*=0.014), lower for PIV/AdV(OR=0.48, 95% CI: 0.25–0.89, *p*=.024), and comparable for the remaining pairs including PIV/RV/EV, the most common co-detection(OR=1.18, 95% CI: 0.86–1.58, *p*=0.27; **Figure 1**; **Table 2**).

Table 2.

**Figure:**
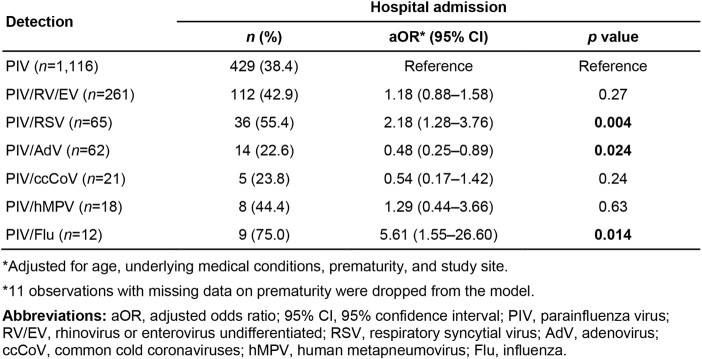

Multivariable logistic regression model of hospitalization in U.S. children with PIV-only detection and those with PIV co-detection with one other respiratory virus, NVSN, 2016–2020.

**Conclusion:**

The association between PIV co-detection and odds of hospitalization was virus-specific for children < 2 years old. Further investigation is warranted to determine if these findings are driven by RSV and influenza's impact on illness severity or if they are indicative of a potential virus–virus interaction that exacerbates PIV disease.

**Disclosures:**

**Janet A. Englund, MD**, Ark Biopharma: Advisor/Consultant|AstraZeneca: Advisor/Consultant|AstraZeneca: Grant/Research Support|GlaxoSmithKline: Grant/Research Support|Meissa Vaccines: Advisor/Consultant|Merck: Grant/Research Support|Moderna: Advisor/Consultant|Moderna: Grant/Research Support|Pfizer: Advisor/Consultant|Pfizer: Grant/Research Support|Sanofi Pasteur: Advisor/Consultant **John V. Williams, MD**, Merck: Grant/Research Support|Quidel: Board Member **Marian G. Michaels, MD, MPH**, Merck: Grant/Research Support|Viracor: Grant/Research Support **Elizabeth P. Schlaudecker, MD, MPH**, Pfizer: Grant/Research Support|Sanofi Pasteur: Advisor/Consultant **Mary A. Staat, MD, MPH**, CDC: Grant/Research Support|Cepheid: Grant/Research Support|Merck: Grant/Research Support|NIH: Grant/Research Support|Pfizer: Grant/Research Support|Up-To-Date: Honoraria **Pedro A. Piedra, MD**, Ark Bioscience: Advisor/Consultant|Ark Bioscience: Grant/Research Support|GSK: Grant/Research Support|Icosavax: Advisor/Consultant|Icosavax: Grant/Research Support|Mapp Biologics: Grant/Research Support|Meissa Vaccines: Grant/Research Support|Moderna: Advisor/Consultant|Novavax: Advisor/Consultant|Novavax: Grant/Research Support|Sanofi-Pasteur: Grant/Research Support|Shionogi: Advisor/Consultant|Shionogi: Grant/Research Support|Takeda: Advisor/Consultant **Rangaraj Selvarangan, BVSc, PhD, D(ABMM), FIDSA, FAAM**, Abbott: Honoraria|Altona Diagnostics: Grant/Research Support|Baebies Inc: Advisor/Consultant|BioMerieux: Advisor/Consultant|BioMerieux: Grant/Research Support|Bio-Rad: Grant/Research Support|Cepheid: Grant/Research Support|GSK: Advisor/Consultant|Hologic: Grant/Research Support|Lab Simply: Advisor/Consultant|Luminex: Grant/Research Support **Natasha B. Halasa, MD, MPH**, Merck: Grant/Research Support|Quidell: Grant/Research Support|Quidell: donation of kits|Sanofi: Grant/Research Support|Sanofi: vaccine support **Geoffrey A. Weinberg, MD**, Merck & Co: Honoraria

